# Characterization of Desmoglein 3 (DSG3) as a Sensitive and Specific Marker for Esophageal Squamous Cell Carcinoma

**DOI:** 10.1155/2022/2220940

**Published:** 2022-02-24

**Authors:** Zhikai Chi, Jyoti Balani, Purva Gopal, Suntrea Hammer, Cheryl M. Lewis, Lan Peng

**Affiliations:** ^1^Department of Pathology, The University of Texas Southwestern Medical Center, Dallas, Texas 75390, USA; ^2^Simmons Comprehensive Cancer Center, The University of Texas Southwestern Medical Center, Dallas, TX 75390, USA

## Abstract

Although P40 and P63 are both sensitive and specific for routine esophageal squamous cell carcinoma (SCC) diagnosis, we recently showed that P40 and P63 immunoreactivities were significantly lower in well-differentiated SCC than those in higher grade tumors. Therefore, a novel esophageal SCC marker, ideally performing better in well-differentiated SCC, is still needed. We characterized desmoglein 3 (DSG3) immunohistochemistry in esophageal SCC, esophageal adenocarcinoma, small-cell lung carcinoma, and large B-cell lymphoma, alongside P40 and CK5/6. The World Health Organization classification was used to grade tumors as well-differentiated (WD), moderately differentiated (MD), or poorly differentiated (PD). There were 20 WD, 26 MD, and 17 PD components among 39 esophageal SCC cases. All esophageal SCC components showed significant DSG3 immunoreactivity (mean, 80%; range, 30%–100%), and the proportions of DSG3 immunoreactive cells were higher in the WD and MD components than in the PD components. No esophageal adenocarcinoma cases showed more than 10% DSG3 immunoreactivity with only weak cytoplasmic staining. With a 5% immunoreactivity cutoff, DSG3 positivity was 100% in all 63 SCC components, 18% in adenocarcinoma cases, and 0% in small-cell lung carcinoma or large B-cell lymphoma cases. The overall DSG3 specificity was 94%. To the best of our knowledge, this is the first study to characterize DSG3 as a sensitive and specific marker for esophageal SCC.

## 1. Backgrounds

Esophageal squamous cell carcinoma (SCC) is an epithelial malignancy displaying squamous differentiation with keratinocyte-type cells, intercellular bridges, or intracytoplasmic keratinization [1, 2]. The distinction between esophageal SCC and adenocarcinoma has become critical for recently developed target therapy and immunotherapy. Immunohistochemistry (IHC) was an important part of the diagnostic workup for esophageal SCC, especially in the small biopsies with limited tissue [3–6]. In pathology practice, P40/P63 (nuclear staining) and CK5/6 (cytoplasmic staining) were commonly used as lineage markers for squamous differentiation [7–11]. The tumor protein P63 belonged to the P53 transcription factor family [12–15]. Due to alternative mRNA splicing, P63 protein can be expressed in two main isoforms as TAp63 and deltaNp63, which can both be recognized by the P63/4A4 antibody, while the P40/BC28 antibody can only recognize the deltaNp63 isoform [16–19]. Although P40 and P63 were sensitive and specific markers for routine esophageal SCC diagnosis, we recently showed that their immunoreactivities were significantly lower in well-differentiated SCC than those in higher grade tumors [20]. Therefore, a novel esophageal SCC marker, ideally performing better in well-differentiated SCC, is still needed.

Desmoglein 3 (DSG3) belongs to the cadherin superfamily and is a part of the adhesion protein complex of desmosomes; DSG3 has been shown to regulate cell adhesion, proliferation, differentiation, morphogenesis, and migration [21–26]. It can also function as an oncogene and facilitate cancer growth and invasion through protein kinase C or DSG3-plakoglobin-TCF/LEF pathways [27]. Indeed, DSG3 has been previously evaluated as a diagnostic marker of squamous differentiation in lung and thymus tumor tissues [28–31]. However, the immunohistochemical analysis of DSG3 in esophageal SCC has not been reported yet. The purpose of this study was to determine the sensitivity and specificity of DSG3 immunostaining and to compare DSG3 with the other SCC markers such as P40 and CK5/6.

## 2. Methods

### 2.1. Study Design and Histomorphological Analysis

This study was reviewed and approved by the Institutional Review Board of the University of Texas Southwestern Medical Center and the Parkland Health and Hospital System. Thirty-nine cases registered in the surgical pathology database of Clements University Hospital and Parkland Health and Hospital System during 2010-2019 that met the diagnostic criteria of esophageal SCC were analyzed in this study, along with samples from 22 esophageal adenocarcinoma cases, 20 small-cell lung carcinoma cases, and 20 large B-cell lymphoma cases during the same period. Clinical information was extracted electronic medical records. The tumor site was defined as the distance of the incisors including upper (<25 cm), mid (25-30 cm), and distal (>30 cm) esophagus. Esophageal SCC and adenocarcinoma cases were graded as well-differentiated (WD), moderately differentiated (MD), or poorly differentiated (PD) according to [1]. Cases involving both squamous and glandular histological features were excluded.

### 2.2. Immunohistochemical Analysis

The primary antibodies used were anti-human P40 (BC28, predilute, Ventana), anti-human CK5/6 (D5/16B4, predilute, Ventana), and anti-human DSG3 (CM419C, 1 : 100, Biocare). Immunohistochemical staining for P40 and CK5/6 was described as before [20]. Briefly, formalin-fixed, paraffin-embedded, 4 *μ*m tissue sections were used with the Ventana Benchmark automated immunostainer (Ventana, Tucson, AZ) and OptiView DAB detection kit. Antigen retrieval time was 32 minutes for P40 and CK5/6. Immunohistochemical stain for DSG3 was performed on a Dako Autostainer Link 48 system. The slides were baked for 20 minutes at 60°C, deparaffinized, hydrated, and then incubated with Proteinase K (Dako, S3020) for 16 minutes, followed by a peroxidase block and then an antibody incubation for 60 minutes. The staining was visualized using the EnVision+ System-HRP visualization system (Agilent, K4009).

P40 and CK5/6 IHC were interpreted based on nuclear (P40) or cytoplasmic (CK5/6) staining and the proportion of immunoreactive cells. DSG3 IHC was interpreted based on distinctly membranous staining and the proportion of immunoreactive cells. Immunoreactive cells were further scored for intensity (none, weak, or strong). “None” was defined as no immunoreactivity; “weak” was defined as significantly less intense immunoreactivity than that observed for squamous basal cells (internal control), while “strong” was defined as equal or more intense immunoreactivity than that observed for squamous basal cells (internal control). Individual cases or components were marked as positive (≥5%) versus negative (<5%) depending on the proportion of immunoreactive tumor cells with any intensity. CK5/6 immunostain was not available in one SCC case with the MD component only. Representative fields were selected and imaged at 100x or 200x magnification.

### 2.3. Statistical Analysis

The *χ*^2^ test was used to analyze categorical data. One-way analysis of variance (posttest: Tukey's multiple comparison test) was used for continuous data. Statistical significance is considered when *p* is less than 0.05.

## 3. Results

### 3.1. Demographics and Clinicopathological Features

The 39 patients with esophageal SCC had an average age of 67 years (range: 37–91 years); there were 30 males among this patient population (77%) (Table 1). There were 33 biopsies, 5 resections, and 1 endoscopic mucosal resection. All 5 patients who underwent resection received neoadjuvant treatments. The adenocarcinoma group consisted of 22 patients with an average age of 68 years (range: 56-84 years), including 17 males (77%). There were 19 biopsies and 3 resections. Among the 3 resection cases, 2 patients received neoadjuvant treatments. There were no significant differences in the average age or sex ratio between the SCC and adenocarcinoma cases (*p* > 0.05). Further, the small-cell lung carcinoma group consisted of 20 patients with an average age of 73 years (range: 56-91 years), including 9 males (45%), while the large B-cell lymphoma group consisted of 20 patients with an average age of 62 years (range: 26-85 years), including 9 males (45%).

### 3.2. Differentiation Grades in Esophageal Squamous Cell Carcinoma and Adenocarcinoma

Esophageal SCC often exhibited mixed intratumoral differentiation. Among the 39 esophageal SCC cases, 4 showed only the WD component, 9 showed only the MD component, 7 showed only the PD component, 9 showed WD+MD components, 2 showed WD+PD components, 3 showed MD+PD components, and 5 showed WD+MD+PD components. In total, there were 20 WD, 26 MD, and 17 PD components (Table 1). In addition, one patient with a single MD squamous component also showed a minor small-cell carcinoma component. Although only the highest grade was assigned to each esophageal SCC in pathology practice, given the morphological heterogeneity that was present in some tumors, we evaluated the individual differentiation component(s) within the same tumor to correlate the immunoprofiles with differentiation grades. Among the 22 adenocarcinoma cases, 7 were graded as MD and 15 as PD. Consequently, the immunoprofiles were evaluated according to the entire adenocarcinoma rather than the individual differentiation component as in the case of SCC.

### 3.3. DSG3 Immunoreactivity and Positivity in Esophageal Squamous Cell Carcinoma

**Figure 1:**
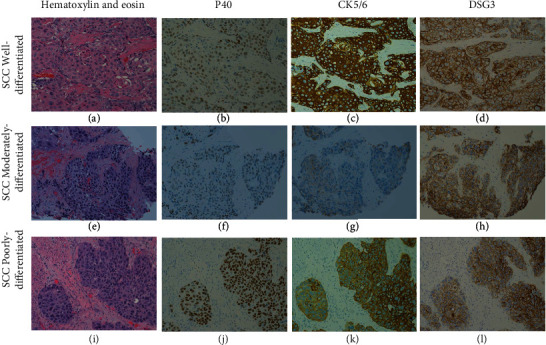
Representative images from one esophageal squamous cell carcinoma (SCC) case: (a–d) well-differentiated component; (e–h) moderately differentiated component; (i–l) poorly differentiated component; (a, e, i) hematoxylin and eosin staining; (b, f, j) P40; (c, g, k) CK5/6; (d, h, l) DSG3. Magnification = 200x (a–l).

All esophageal SCC components showed DSG3 immunoreactivity (mean, 80%; range, 30%–100%); the proportion of DSG3 immunoreactive cells was higher in the WD and MD components than that in the PD components (mean, 87% vs. 85% vs. 63%, respectively; *p* < 0.001) (Table 2). WD and MD components showed stronger DSG3 intensities than that observed for the PD component (percentage of components with strong intensity, 100% vs. 96% vs. 65%, respectively; *p* < 0.001) with 89% (56/63) of all components showing strong intensities. The distinct membranous staining pattern was retained in DSG3 immunoreactive SCC cells (Figure 1). For comparison, the proportions of P40 immunoreactive cells were significantly lower in the WD components than those in the MD or PD components, while the proportions of CK5/6 immunoreactive cells were similar among differentiation components (Table 2). Notably, the single small-cell carcinoma component showed 0% DSG3 immunoreactivity.

**Figure 2:**
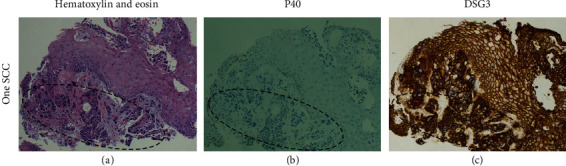
Images from one esophageal squamous cell carcinoma (SCC) case: (a) hematoxylin and eosin staining, (b) P40, and (c) DSG3. Carcinoma cells were circled out by dashed lines. Magnification = 100x (a–c).

Using a 5% immunoreactivity cutoff, the positivity for WD SCC components was 100% for DSG3 and CK5/6, but 95% (19/20) for P40; for MD and PD components, all three immunostaining procedures achieved 100% positivity (Table 3). Overall, the positivity for all SCC components was 98% (62/63) for P40 and 100% for both CK5/6 and DSG3 (Table 3). One patient with only WD component showed negative P40 (2% immunoreactivity) but positive DSG3 (100% strong immunoreactivity) (Figure 2).

### 3.4. DSG3 Immunoreactivity and Positivity in Esophageal Adenocarcinoma, Small-Cell Lung Carcinoma, and Large B-Cell Lymphoma

In esophageal adenocarcinoma cases, the proportion of DSG3 immunoreactive cells was much lower and showed no significant difference between MD and PD cases (mean, 2% vs. 2%, respectively; *p* > 0.05) (Table 4). Notably, none of the adenocarcinoma cases showed more than 10% DSG3 immunoreactivity which was weak cytoplasmic staining rather than the distinct membranous staining seen in esophageal SCC. All small-cell lung carcinoma and large B-cell lymphoma cases showed zero DSG3 immunoreactivity (Table 4). Using a 5% immunoreactivity cutoff, the positivity for 7 MD and 15 PD adenocarcinoma cases was 14% (1/7) and 20% (3/15), respectively (Table 4). In summary, the DSG3 positivity rate was 18% (4/22) for adenocarcinoma cases and 0% for both small-cell lung carcinoma and large B-cell lymphoma cases (Table 4). The overall DSG3 specificity is 94% (58/62).

## 4. Discussion

In this study, significant DSG3 immunoreactivities were observed in all three differentiation components in esophageal SCC. The proportions of DSG3 immunoreactive cells were significantly higher in the WD and MD components than those in the PD components. As we have previously reported, P40 immunoreactivities were significantly lower in WD SCC than those in higher grade tumors, which potentially represents a diagnostic pitfall when interpreting P40 IHC in small biopsy samples [20]. Indeed, in our case series, we encountered one patient with the WD component showing negative P40 (2% immunoreactivity) but positive DSG3 (100% strong immunoreactivity). In addition, DSG3 IHC showed distinct membranous staining patterns with strong intensities in 89% SCC components. Although CK5/6 IHC showed similar immunoreactivities in esophageal SCC, DSG3 IHC was easier to interpret due to its distinct membranous staining patterns. These findings indicated that DSG3 can act as a highly sensitive and easy-to-interpret marker for esophageal SCC. Although histomorphological features are usually sufficient to diagnose esophageal SCC, if pathologists deem IHC to be necessary, we recommend a combination of P40/P63 (nuclear stain) with either DSG3 (membranous stain) or CK5/6 (cytoplasmic stain) to better cover all the differentiation components of esophageal SCC.

Distinction of esophageal SCC from other morphological mimics has become critical in the era of widespread application of individualized target therapies. Using a 5% immunoreactivity cutoff, the DSG3 positivity was 18% for esophageal adenocarcinoma, 0% for small-cell lung carcinoma, and 0% for large B-cell lymphoma. The overall DSG3 specificity was 94%. In pathology practices, the most important differentiation diagnosis for esophageal SCC is esophageal adenocarcinoma; therefore, 18% of DSG3 positivity appeared to be not ideal at first look. However, none of the adenocarcinoma cases had more than 10% DSG3 immunoreactivity, and they all showed weak cytoplasmic staining patterns rather than the distinct membranous patterns seen in esophageal SCC. If the immunoreactivity cutoff was to be raised to 10%, DSG3 positivity would have been 0% for esophageal adenocarcinoma, but still 100% for esophageal SCC. Therefore, our findings indicated that DSG3 is a highly specific marker to differentiate esophageal SCC from adenocarcinoma. Additionally, distinction from small-cell carcinoma is also important. However, primary esophageal small-cell carcinoma was rare that we were not able to identify such cases for our study period. Interestingly, we did have one esophageal SCC case with a minor small-cell carcinoma component, and this minor small-cell carcinoma component showed 0% DSG3 immunoreactivity. Furthermore, lung small-cell carcinoma could potentially metastasize to lymph nodes adjacent to the esophagus and present as an esophageal mass, wherein the differential diagnosis between lung small-cell carcinoma and esophageal SCC would arise. Therefore, our results of 0% DSG3 immunoreactivity in lung small-cell carcinoma were helpful in this aspect.

Lastly, DSG3 can function as an oncogene and facilitate cancer growth and invasion through protein kinase C or DSG3-plakoglobin-TCF/LEF pathways [27]. Subsequently, DSG proteins have been shown to be potential therapeutic targets in SCC from skin, head and neck, and lung [32–34]. Anti-DSG3 antibody generated without pathogenic activity of pemphigus vulgaris showed high antibody-dependent cell cytotoxicity (ADCC) against DSG3-expressing lung SCC [33]. Now, for the first time, our studies provided detailed characterization of DSG3 expression in esophageal SCC, which paved the way for future research on the therapeutic applications of anti-DSG3 antibody in esophageal SCC patients.

There are several limitations to the current study. First, due to the aggressive nature of esophageal SCC, most patients' tumors in this study were considered locally advanced or unresectable. As a result, the majority of specimens were biopsies rather than resection specimens, which potentially led to either overestimation or underestimation of the heterogeneity of DSG3 expression in the tumor. Second, all our cases, which had already been finalized as SCC, were retrospectively submitted for immunostaining; therefore, the morphology was convincing, and in many instances, squamous differentiation had previously been supported by other immunostains. Thirdly, the number of cases included in our study is still small due to the relatively low prevalence of esophageal SCC in the USA. Lastly, all the cases came from closely related two hospitals; thus, a patient population selection bias could not have been avoided.

## 5. Conclusion

This is the first study to show DSG3 as a sensitive and specific marker for esophageal SCC. The findings of this study provide evidence regarding the optimized choice of squamous differentiation markers in routine pathology practice as well as detailed characterization of DSG3 expression in esophageal SCC.

## Figures and Tables

**Table 1:** Clinicopathological features of patients with esophageal squamous cell carcinoma.

Age (years) (mean, range)	67 (37-91)	

Gender (*n* = 39)	Male (*n* = 30)	77%
	Female (*n* = 9)	23%

Procedures (*n* = 39)	Biopsy (*n* = 33)	
	Resection (*n* = 5)	
	Endoscopic mucosal resection (*n* = 1)	

Tumor site (*n* = 39)	Upper esophagus (*n* = 14)	36%
	Mid esophagus (*n* = 5)	13%
	Distal esophagus (*n* = 10)	26%
	Mid and distal esophagus (*n* = 10)	26%

Differentiation components	Well-differentiated components (*n* = 20)	
	Moderately differentiated components (*n* = 26)	
	Poorly differentiated components (*n* = 17)	
	Small-cell carcinoma component (*n* = 1)	

**Table 2:** Proportion and intensity of immunoreactive cells in esophageal squamous cell carcinoma.

	P40 proportion (average, range)					CK5/6 proportion (average, range)					DSG3 proportion (average, range)				
SCC WD components	38% (2-70%, *n* = 20)^∗∗∗^					81% (30-100%, *n* = 20)					87% (60-100%, *n* = 20)^∗∗∗^				
SCC MD components	71% (10-90%, *n* = 26)					87% (20-100%, *n* = 25)^∗^					85% (40-100%, *n* = 26)				
SCC PD components	90% (90-95%, *n* = 17)					73% (20-100%, *n* = 17)					63% (30-90%, *n* = 17)				
SCC all components	66% (2-95%, *n* = 63)					81% (20-100%, *n* = 62)					80% (30-100%, *n* = 63)				
	P40 proportion range (*n*)					CK5/6 proportion range (*n*)					DSG3 proportion range (*n*)				
	<5%	5-25%	26-50%	51-75%	>75%	<5%	5-25%	26-50%	51-75%	>75%	<5%	5-25%	26-50%	51-75%	>75%
SCC WD components	1	5	11	3	0	0	0	4	1	15	0	0	0	2	18
SCC MD components	0	1	5	4	16	0	0	1	2	22	0	0	2	0	24
SCC PD components	0	0	0	0	17	0	0	2	3	12	0	0	8	0	9
	P40 intensity (number, percentage)					CK5/6 intensity (number, percentage)					DSG3 intensity (number, percentage)				
	None		Weak	Strong		None		Weak	Strong		None		Weak	Strong	
SCC WD components	0		4/20 (20%)	16/20 (80%)		0		1/20 (5%)	19/20 (95%)		0		0	20/20 (100%)^∗∗∗^	
SCC MD components	0		2/26 (8%)	24/26 (92%)		0		2/25 (8%)	23/25 (92%)		0		1/26 (4%)	25/26 (96%)	
SCC PD components	0		0	17/17 (100%)		0		6/17 (35%)	11/17 (65%)		0		6/17 (35%)	11/17 (65%)	

Abbreviations: SCC: squamous cell carcinoma; WD: well-differentiated; MD: moderately differentiated; PD: poorly differentiated. ^∗∗∗^*p* < 0.001, P40 or DSG3 proportions in SCC WD vs. MD vs. PD components, one-way ANOVA. ^∗∗∗^*p* < 0.001, DSG3 strong intensity in SCC WD vs. MD vs. PD components, *χ*^2^ test. ^∗^CK5/6 immunostain is not available in one SCC case with MD component only.

**Table 3:** Percentage of positive esophageal squamous cell carcinoma components (≥5% immunoreactive cells).

	P40	CK5/6	DSG3
SCC WD components	95% (19/20)	100% (20/20)	100% (20/20)
SCC MD components	100% (26/26)	100% (25/25)^∗^	100% (26/26)
SCC PD components	100% (17/17)	100% (17/17)	100% (17/17)
SCC all components	98% (62/63)	100% (62/62)	100% (63/63)

Abbreviations: SCC: squamous cell carcinoma; WD: well-differentiated; MD: moderately differentiated; PD: poorly differentiated. ^∗^CK5/6 immunostain is not available in one SCC case with MD component only.

**Table 4:** DSG3 immunostaining in esophageal adenocarcinoma, small-cell lung carcinoma, and large B-cell lymphoma.

	DSG3 proportion (average, range)				
Adenocarcinoma MD cases (*n* = 7)	2% (0-10%)				
Adenocarcinoma PD cases (*n* = 15)	2% (0-10%)				
Small-cell lung carcinoma (*n* = 20)	0% (0-0%)				
Large B-cell lymphoma (*n* = 20)	0% (0-0%)				
	DSG3 proportion range (*n*)				
	<5%	5-25%	26-50%	51-75%	>75%
Adenocarcinoma MD cases (*n* = 7)	6	1	0	0	0
Adenocarcinoma PD cases (*n* = 15)	12	3	0	0	0
	DSG3 intensity (number)				
	None		Weak	Strong	
Adenocarcinoma MD cases (*n* = 7)	4		3	0	
Adenocarcinoma PD cases (*n* = 15)	10		5	0	
	Percentage of positive cases (≥5% immunoreactive cells)				
Adenocarcinoma MD cases (*n* = 7)	14% (1/7)				
Adenocarcinoma PD cases (*n* = 15)	20% (3/15)				
Adenocarcinoma, all cases (*n* = 22)	18% (4/22)				
Small-cell lung carcinoma (*n* = 20)	0% (0/20)				
Large B-cell lymphoma (*n* = 20)	0% (0/20)				

Abbreviations: MD: moderately differentiated; PD: poorly differentiated.

## Data Availability

The data used to support the findings of this study are available from the corresponding author upon request.
